# The SURV1VE trial—sustained inflation and chest compression versus 3:1 chest compression-to-ventilation ratio during cardiopulmonary resuscitation of asphyxiated newborns: study protocol for a cluster randomized controlled trial

**DOI:** 10.1186/s13063-019-3240-8

**Published:** 2019-02-19

**Authors:** Georg M. Schmölzer, Gerhard Pichler, Anne Lee Solevåg, Caroline Fray, Sylvia van Os, Po-Yin Cheung, on behalf of the SURV1VE trial collaborators

**Affiliations:** 1 Centre for the Studies of Asphyxia and Resuscitation, Neonatal Research Unit, Royal Alexandra Hospital, 10240 Kingsway Avenue NW, Edmonton, AB T5H 3V9 Canada; 2 Department of Pediatrics, University of Alberta, Edmonton, Canada; 3 Division of Neonatology, Department of Pediatrics, Medical University Graz, Graz, Austria; 4 Department of Pediatrics and Adolescent Medicine, Akershus University Hospital, Lørenskog, Norway

## Abstract

**Background:**

The need for cardiopulmonary resuscitation (CPR) is often unexpected, and the infrequent use of CPR in the delivery room (DR) limits the opportunity to perform rigorous clinical studies to determine the best method for delivering chest compression (CC) to newborn infants. The current neonatal resuscitation guidelines recommend using a coordinated 3:1 compression-to-ventilation (C:V) ratio (CC at a rate of 90/min and ventilations at a rate of 30/min). In comparison, providing CC during a sustained inflation (SI) (CC + SI) significantly improved hemodynamics, minute ventilation, and time to return of spontaneous circulation (ROSC) compared to 3:1 C:V ratio in asphyxiated piglets. Similarly, a small pilot trial in newborn infants showed similar results. Until now no study has examined different CC techniques during neonatal resuscitation in asphyxiated newborn infants in the DR. To date, no trial has been performed to directly compare CC + SI and 3:1 C:V ratio in the DR during CPR of asphyxiated newborn infants.

**Methods:**

This is a large, international, multi-center, prospective, unblinded, cluster randomized controlled trial in asphyxiated newborn infants at birth. All term and preterm infants > 28^+ 0^ by best obstetrical estimate who require CPR at birth due to bradycardia (< 60/min) or asystole are eligible. The primary outcome of this study is to compare the time to ROSC in infants born > 28^+ 0^ weeks’ gestational age with bradycardia (< 60/min) or asystole immediately after birth who receive either CC + SI or 3:1 C:V ratio as the CPR strategy.

**Discussion:**

Morbidity and mortality rates are extremely high for newborns requiring CC. We believe the combination of simultaneous CC and SI during CPR has the potential to significantly improve ROSC and survival. In addition, we believe that CC + SI might improve respiratory and hemodynamic parameters and potentially minimize morbidity and mortality in newborn infants. In addition, this will be the first randomized controlled trial to examine CC in the newborn period.

**Trial registration:**

ClinicalTrials.gov, NCT02858583. Registered on 8 August 2016

**Electronic supplementary material:**

The online version of this article (10.1186/s13063-019-3240-8) contains supplementary material, which is available to authorized users.

## Background and rationale

The need for cardiopulmonary resuscitation (CPR) is often unexpected, and the infrequent use of CPR in the delivery room (DR) limits the opportunity to perform rigorous clinical studies to determine the best method for delivering chest compression (CC) to newborn infants [1–5]. The main cause of cardiovascular collapse in newborn infants is asphyxia, which makes newborn infants distinctively different from the adult population [6]. The International Liaison Committee on Resuscitation and the American Academy of Pediatrics/American Heart Association Neonatal Resuscitation Program acknowledge this difference [7, 8]. The current neonatal resuscitation guidelines recommend using a coordinated 3:1 compression-to-ventilation (C:V) ratio (CC at a rate of 90/min and ventilations at a rate of 30/min) [7, 8]. However, due to the lack of clinical data from newborn infants, the guidelines are based on data from adults and animal studies [7, 8]. Such data may not be wholly applicable to the neonatal population because the most common cause of cardiovascular collapse in the adult is ventricular fibrillation, not asphyxia [9, 10].

Several piglet studies have investigated various C:V ratios in asphyxia-induced cardiac arrest. These studies suggest that during neonatal CPR different C:V ratios do not improve outcomes [9–14]. Furthermore, continuous CC with asynchronous ventilations (CCaV) compared to 3:1 C:V CPR in a piglet model of neonatal asphyxia had similar results for time to return of spontaneous circulation (ROSC) (114 and 143 s for CCaV and 3:1, respectively) and survival (3/8 and 6/8, respectively), suggesting that continuous CC alone does not rescue ROSC [15].

In asphyxiated piglets, Schmölzer et al. reported that passive ventilation during CC, achieved by providing CC during a sustained inflation (SI) (CC + SI), significantly improved hemodynamics, minute ventilation, and time to ROSC compared to 3:1 C:V ratio [16]. Increased intrathoracic pressure and improved oxygenation during CC + SI might have contributed to the improved outcomes. Chandra et al. provided ventilation at high airway pressure while simultaneously performing CC in an animal model and demonstrated increased carotid flow without compromising oxygenation [17]. Further, studies in preterm lambs have demonstrated that a SI also increases intrathoracic pressure without impeding blood flow [18–21]. However, the study by Schmölzer et al. used a CC rate of 120/min (in the CC + SI group), which is higher than the currently recommended CC rate of 90/min [7, 8], which could have added to the improved outcomes. A further randomized animal trial compared CC + SI using a CC rate of 90/min versus 120/min and reported similar time to ROSC, survival rates, and respiratory parameters during CPR [22]. During CC carotid blood flow, mean arterial pressure, and percent change in ejection fraction and cardiac output were higher in the CC + SI 90/min group compared to CC + SI 120/min [22].

These findings support that higher CC rates [23] do not improve systemic perfusion and that the current recommendation of 90 CC/min is sufficient to achieve systemic perfusion. Also, CC + SI 90/min compared to 3:1 C:V ratio in a porcine model of neonatal resuscitation reported a significant reduction in median (interquartile range (IQR)) time to ROSC, 34 (28–156) s versus 210 (72–300) s (*p* = 0.05), and less use of supplementary oxygen during CPR (*p* = 0.03) and epinephrine (*p* = 0.32) in the CC + SI 90/min group [24]. In addition, improved respiratory and hemodynamic parameters were observed in the CC + SI 90/min group versus the 3:1 C:V group. Similarly, a recent trial using a transitional newborn lambs model reported that CC + SI is feasible at birth with similar ROSC times compared to 3:1 C:V [25], which might have been due to the shape of the sheep’s chest [9, 10]. Further piglet studies examined 20s versus 60s of SI during CC + SI with similar time to ROSC [26], with an optimal inflation pressure during CC + SI of 25 cm H_2_O [27]. Most recently, a pilot trial in preterm infants (< 32 weeks’ gestation) showed similar results [28]. Overall, the mean (standard deviation (SD)) time to ROSC was significantly shorter in the CC + SI group, with 31 (9) s compared to 138 (72) s in the 3:1 C:V group (*p* = 0.011). Overall, 0/5 in the CC + SI group and 1/5 in the 3:1 C:V group received epinephrine.

Successful resuscitation from cardiac arrest or severe bradycardia requires the delivery of high-quality CC as well as adequate ventilation. However, no study has examined different CC techniques during neonatal resuscitation in asphyxiated newborn infants in the DR. To date, no trial has been performed to directly compare CC + SI and 3:1 C:V ratio in the DR during CPR of asphyxiated newborn infants.

### Aims

The primary objective of this study is to compare the time to ROSC in infants born > 28^+ 0^ weeks’ gestational age (GA) with bradycardia (< 60/min) or asystole immediately after birth who receive either CC + SI or 3:1 C:V ratio as the CPR strategy.

## Methods/design

**Fig. 1:**
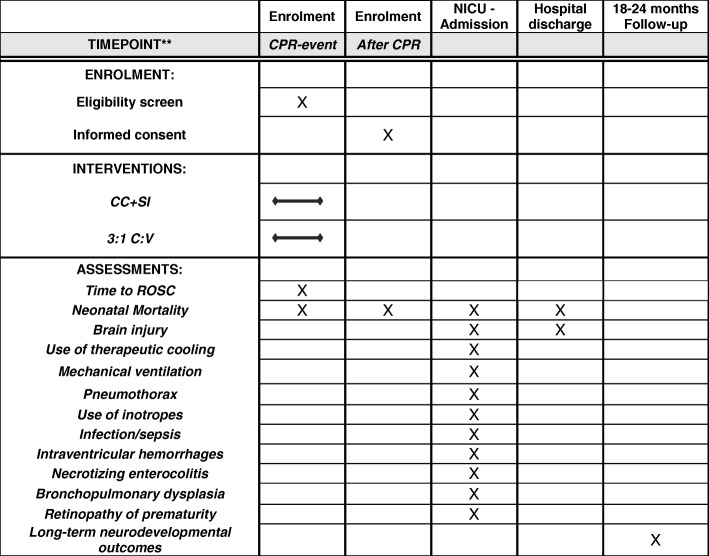
Example template of recommended content for the schedule of enrollment, interventions, and assessments*

This is a large, international, multi-center, prospective, unblended, cluster randomized controlled trial in asphyxiated newborn infants at birth. The trial is registered at ClinicalTrials.gov with the identifier NCT02858583. The analysis will be conducted using an “intention-to-treat” approach, and the trial will be reported using the Consolidated Standards of Reporting Trials (CONSORT) extension for cluster trials [29]. The Standard Protocol Items: Recommendations for Interventional Trials (SPIRIT) checklist for this study is provided as Additional file 1. An example template of recommended content for the schedule of enrollment, interventions, and assessments is presented in Fig. 1.

### Population

Patients will be recruited in this international, multi-center trial in 25 tertiary level delivery hospitals located in Canada, the USA, Australia, Asia, China, and Europe.

#### Inclusion criteria

The inclusion criteria refer to all term and preterm infants > 28^+ 0^ by best obstetrical estimate who require CPR at birth due to bradycardia (< 60/min) or asystole.

#### Exclusion criteria

The exclusion criteria are as follows:

1. Refusal of informed consent
2. Known major congenital anomalies that might have an adverse effect on breathing or ventilation (e.g., congenital diaphragmatic hernia) or congenital heart disease requiring intervention in the neonatal period
3. Infants born to mothers who are unable to give informed consent for their medical care and who do not have a surrogate guardian.

### Recruitment

As the need for CPR in the DR is often unexpected and infrequent, obtaining informed consent prior to the study (e.g., during the antenatal period) is not feasible. Therefore, the study will use a combination of waiver of consent and deferred consent model at all sites. Written consent will be sought from the parents of these infants as soon as possible after the birth so that acquired data can be utilized for research [30]. Currently 20 clinical sites have applied for ethics, and the data coordinating center has received Ethics Board approval at the time of this submission; all remaining sites will obtain Ethics Board approval prior to initiating subject recruitment.

### Randomization

At the beginning of the trial, all participating sites will be randomized by the Clinical Research Informatics Core and Biostatistics Core of the Women and Children’s Health Research Institute. Centers will be equally allocated to either CC + SI (“intervention”) or 3:1 C:V ratio (“control”) for the first year. For the second year, after a washout period of 2 months (for training of the other intervention), centers in the “intervention group” will be changed to “control group” centers and vice versa.

### Blinding

The study intervention is unblinded. Each hospital is allocated to one treatment arm, and therefore the CC technique used at each site is known to the clinical team. However, to protect against potential bias in outcome ascertainment, the outcome assessors are unaware of the group allocation. This blinding will be maintained until the data are locked for the final analysis and unblinded.

### Intervention

#### Techniques of resuscitation

**Fig. 2:**
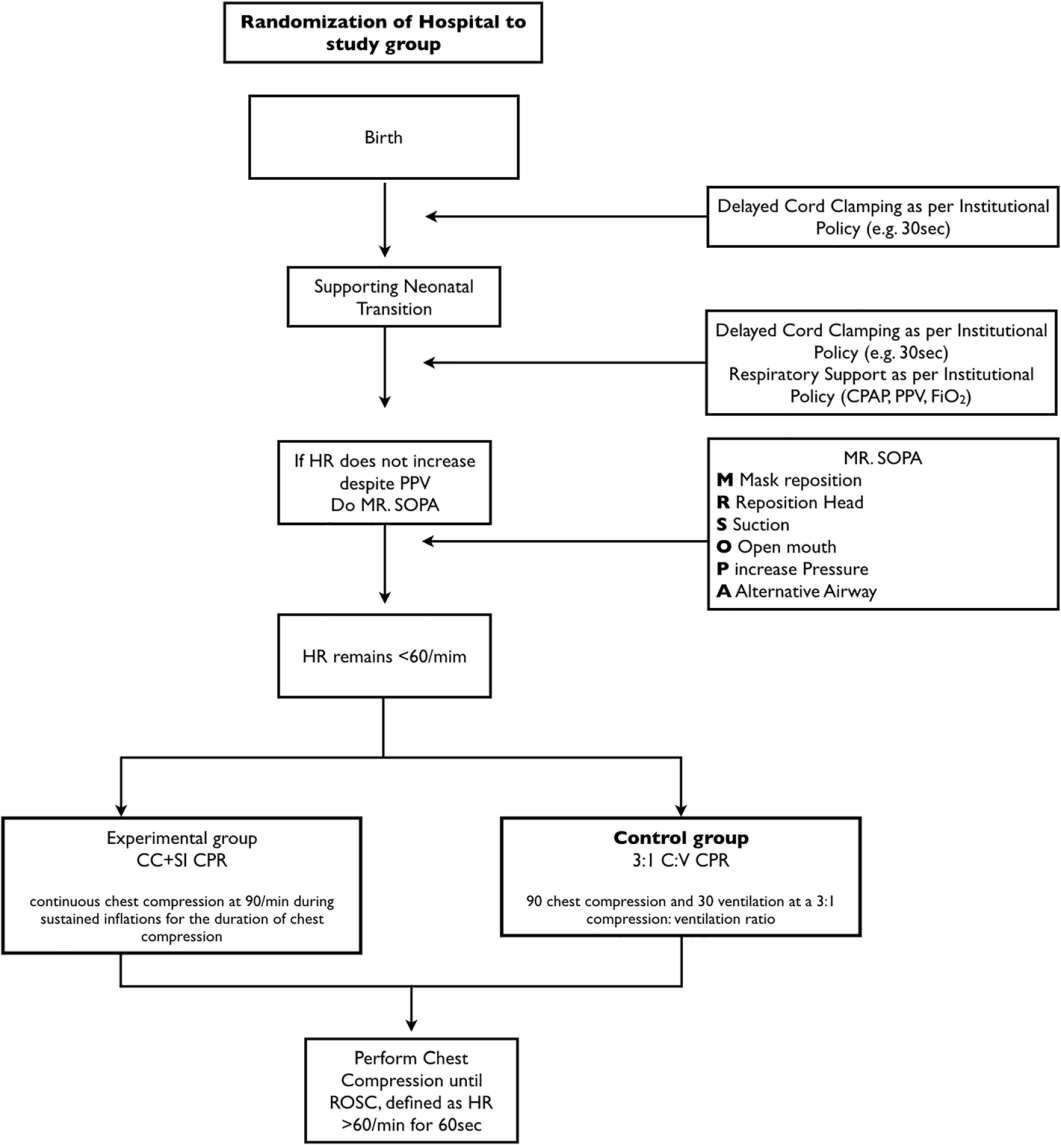
Study flow chart

The following sections describe general resuscitation interventions and the trial intervention (Fig. 2).

#### Constitution of the resuscitation team

The members of the resuscitation team will be site-specific (for example, in Edmonton, Canada the team consists of a Registered Neonatal Nurse (RN), Respiratory Therapist (RT), Pediatric Resident or Neonatal Sub-specialty Resident (Fellow) or Neonatal Nurse Practitioner (NNP) and/or Neonatologist). The most senior team member will act as the team leader, who will manage the protocol and guide the intervention during the trial.

#### Description of general interventions

##### Term infants

The initial steps of the resuscitation will be performed according to the current neonatal resuscitation guidelines [7, 8]. Delayed cord clamping should be performed as per local hospital policy (standard hospital practice guidelines) at participating sites where eligible. Afterwards, dry and stimulate the infant, and open the airway. If the heart rate is < 100/min, or the infant is gasping or has apnea, start positive pressure ventilation (PPV) with 21% oxygen. At the same time consider attaching a pulse oximeter and consider applying electrocardiography. If the heart rate remains < 100/min, perform MR SOPA (*M*ask is tightly applied to the face, *R*e-position the head into the “sniffing” orientation, *S*uction the nares and the pharynx, *O*pen the mouth, *P*ressure of PPV can be increased to a max of 40 cm H_2_O, *A*lternate airway (endotracheal tube or laryngeal mask airway). If the heart rate is < 60/min or remains < 60 despite all steps of MR SOPA including alternate airway, start CC and increase to 100% oxygen on the ventilation device.

##### Preterm infants > 28^+ 0^ weeks’ gestation

The initial steps of the resuscitation will be performed according to the current neonatal resuscitation guidelines [7, 8]. Delayed cord clamping should be performed as per local hospital policy (standard hospital practice guidelines) at participating sites where eligible. Preterm infants (according to local hospital policy and standard hospital practice guidelines) will be either placed (without drying) in a polyethylene bag under radiant heat [31] or dried and placed under radiant heat. A pulse oximeter will be used to monitor oxygen saturation immediately after birth, and oxygen delivery will be guided by published norm values for oxygen saturation in preterm infants [32]. If the heart rate is < 100/min, or the infant is gasping or has apnea, start PPV. Also consider applying electrocardiography at that time. If the heart rate remains < 100/min, perform MR SOPA. If the heart rate is < 60/min or remains < 60 despite all steps of MR SOPA, start CC and increase to 100% oxygen on the ventilation device.

#### Mask ventilation

The clinical team will determine if an infant requires mask ventilation*.* If mask ventilation is required, it should be provided with a T-Piece device (e.g., from Fisher & Paykel, Auckland, New Zealand) or a Giraffe Warmer (GE Health Care, Burnaby, BC, Canada) and an appropriate face mask with default settings for a peak inflation pressure (PIP) of 20–30 cmH_2_O, a positive end-expiratory pressure (PEEP) of 5–8 cm H_2_O, and a gas flow rate of 8–10 L/min using a ventilation rate of 40–60 inflations/min. Initial respiratory support can include initial sustained inflations (instead of standard PPV) as per local hospital policy (standard hospital practice guidelines) [7, 8].

#### Cardiopulmonary resuscitation

If the heart rate is < 60/min or remains < 60/min despite all steps of MR SOPA, start CC. Prior to the start of CC, consider intubation. However, in cases of intubation failure it is possible to start CC during mask ventilation. CC will be performed using the two-thumb encircling hands technique [7, 8]. Epinephrine will be administered according to guidelines either via umbilical vein catheter (0.01 mg/kg per dose — preferred) or via endotracheal tube (0.05 mg/kg to 0.1 mg/kg) every 3–5 min as needed [7, 8]. CC and epinephrine will be continued until ROSC.

#### Determination of ROSC

ROSC will be defined as an increase in heart rate > 60/min for 60s defined by cardiac auscultation.

#### Intervention groups

##### 3:1 C:V group

If the PIP has been increased > 30 cm H_2_O during MR SOPA, the PIP should be decreased to 30 cmH_2_O prior to start of CC. Infants randomized into the 3:1 C:V group will receive CC at a rate of 90/min and ventilations at a rate of 30/min in a 3:1 C:V ratio as recommended in the guidelines (see Fig. 1) [7, 8]. During CC, the inflations should be delivered using a PIP of 25–30 cmH_2_O (as per local hospital policy/standard hospital practice guidelines). The heart rate should be re-evaluated every 60s [7, 8].

##### CC + SI group

If the PIP has been increased > 30 cm H_2_O during MR SOPA, the PIP should be decreased to 30 cmH_2_O prior to the start of CC. Infants randomized into the CC + SI group will receive a SI with a PIP of 25–30 cmH_2_O while receiving CC. CC will be performed at a rate of 90/min. The SI will be delivered over a period of 20s. This will be followed by a PEEP of 5–8 cm water as per local hospital policy (standard hospital practice guidelines) for 1 s. Then the next SI for 20s is started while the CCs are continued. Again, after 20s there will be a 1-s pause with a PEEP, which will be followed by another SI for 20s with continuous CC. After 3 × 20 s CC + SI (a total of 60s) a heart rate assessment should be performed. If the heart rate is > 60/min, continue with standard care as per local hospital policy (standard hospital practice guidelines). If the heart rate remains < 60/min, continue with CC + SI for another 60s (3 × 20 s CC + SI), at which time a further assessment should be performed. If the heart rate remains < 60/min, continue with CC + SI. If CPR is ongoing for 5 min using CC + SI, the clinical team must convert to the standard method of care using the 3:1 C:V ratio.

#### Discontinuing resuscitation

Deciding how long resuscitative efforts should continue in any individual infant will be solely at the discretion of the clinical team in accordance with guidelines and local hospital policy (standard hospital practice guidelines) at the participating sites [7, 8].

#### Recording of physiological parameters

Participating sites with the capability to record physiological parameters and/or video during neonatal resuscitations can include this if time permits prior to delivery [33–37]. Secondary objectives of the study will be to examine changes in physiological parameters and respiratory function during CPR.

#### Primary outcome measure

The primary outcome measure is the time to achieve ROSC defined as a heart rate of > 60/min for 60s defined by cardiac auscultation.

#### Secondary outcome measures

Secondary outcomes among others will include physiological parameters and respiratory function during CPR, neonatal mortality (death within 28 days of life) and morbidities, e.g., brain injury (reported either via magnetic resonance imaging (MRI) or head ultrasound), changes in regional cerebral oxygen saturation using near-infrared spectroscopy (NIRS) monitoring started in the DR and discontinued when clinical stabilization is achieved, results of amplitude integrated electroencephalography (aEEG) monitoring until normalization of background patterns and occurrence of sleep-wake cycling, DR interventions (including use of epinephrine), neonatal intensive care unit (NICU) admission temperature, use of therapeutic cooling, use of mechanical ventilation, pneumothorax, use of inotropes, infection/sepsis, intraventricular hemorrhage, necrotizing enterocolitis, bronchopulmonary dysplasia, retinopathy of prematurity, and long-term neurodevelopmental outcomes.

#### Sample size and power calculation

Our primary outcome measure will be time to ROSC. We hypothesize that the time to ROSC will be reduced in the CC + SI group. A sample size of 208 infants, 104 in each group, would be sufficient to detect a clinically important 33% reduction in time to achieve ROSC using Cox proportional hazards regression with 80% power and a two-tailed alpha error of 0.05. This 33% reduction represents 282 s versus 420 s of CC (based on the database of 30 term infants requiring CC in 2014 and 2015 at the Royal Alexandra Hospital, Edmonton, Canada). To account for cluster randomization, the sample size is multiplied by a design effect of 1.045, so the total sample size is 218 infants, 109 in each group.

#### Data collection and analysis

##### Outcome assessment tools

Outcome assessment will include mortality and morbidity. For mortality, all causes will be recorded. Cerebral injury will be assessed with cerebral MRI or ultrasound performed prior to discharge. If an infant dies prior to any neuroimaging, a request for autopsy should be made. Autopsy could include imaging alone or full autopsy. For morbidity, there will be a case history until discharge or term age (for preterm infants). Ancillary studies include outcomes at 18–24 months of age.

##### Compliance with the protocol

The clinical investigation will be conducted in compliance with this protocol. Modifications to the protocol will not be implemented without agreement from the principal investigators, and relevant ethics committee approval must also be obtained. Investigators are not allowed to deviate from the protocol except as specified above. Any major or safety-related deviations will be recorded and analyzed, and the ethics committees notified. If an investigator refuses to comply with the protocol, he/she will be disqualified.

##### Data collection

Infants will be recruited over a period of 26 months in each participating center. Approximately another 6 months will be required to collect hospital data on all infants enrolled. Resuscitation data will be collected on a standard form (Neonatal Resuscitation Record) that will form part of each infant’s hospital record. The Neonatal Resuscitation Record should be completed by the clinical team attending the resuscitation. Other medical data on each infant will be collected on an electronic Case Report Form (eCRF). The eCRF will be designed in collaboration with the Women and Children’s Health Research Institute, University of Alberta, Edmonton, Canada. Data will be entered into the REDCap database from each site within 1 week after discharge or death of an infant. Long-term follow-up data will be entered within 2 weeks after examination. All information entered into the REDCap database will be used for analysis.

##### Data analysis

The Clinical Research Informatics Core and Biostatistics Core of the Women and Children’s Health Research Institute, University of Alberta, will perform data management and analysis. Additional information about the storage, management, and analysis of study data is available. All analyses will be carried out according to the intention-to-treat principle. Statistical analyses will be performed using IBM SPSS Statistics Ver. 24 (IBM Corp.) and SAS version 9.4 or later (SAS Institute Inc.).

##### Interim analysis

Interim safety analyses will be performed at 10%, 25%, and 50% of enrollment to review the primary outcome ROSC and severe adverse events (SAEs). Thus, three interim analyses will be performed at 22 patients (11 patients per group), 54 (27 patients per group), and 110 (55 patients per group) to evaluate the safety and efficacy of the intervention. At each interim analysis, the posterior probability of the CC + SI arm to reduce time to ROSC by 10% or more compared to the control arm will be calculated. If this probability is less than 0.5, the trial will be stopped for futility. If the posterior probability is greater than 0.98, the Data and Safety Monitoring Board (DSMB) will consider the trial to be stopped for superiority. Since no statistical tests will be performed at interim analyses, the type I error (alpha) does not need adjustment.

##### Final analysis

**Primary analysis**

The primary outcome is the time to achieve ROSC. Data will be analyzed on an intention-to treat basis and will include all randomized participants. A per protocol analysis will also be conducted using the data from the actual allocation of participating infants. A survival analysis will be used to analyze the difference in time to ROSC between the intervention and control groups. To account for cluster randomization, Cox proportional hazards regression with time to ROSC as an outcome and allocation group as an independent variable will be created. Centers will be entered as clusters in the model, and the statistical significance of the allocation group variable will be evaluated. The analysis will be two-sided, and a *p* value < 0.05 will be considered statistically significant.

**Secondary analysis**

The data will be presented as mean (standard deviation, SD) for normally distributed continuous variables and median (interquartile range, IQR) when the distribution is skewed. The clinical characteristics and outcome parameters will be compared using Student’s *t* test for parametric and the Mann-Whitney *U* test for nonparametric comparisons of continuous variables, and χ^2^ for categorical variables. All *p* values will be two-sided, and *p* < 0.05 will be considered statistically significant.

#### Assessment of safety

##### Adverse and serious adverse events

Infants who require CPR in the DR are a very seriously ill patient group. Most adverse events may be of a serious nature with or without the SURV1VE trial intervention, and both intervention groups are expected to have a very high proportion of SAEs. SAEs to be recorded are therefore only mortality within the DR (e.g., did not achieve ROSC or did achieve ROSC but care was withdrawn) or within the NICU (any mortality).

##### Expected adverse events

Adverse events we expect to be related to the application of the treatment guideline include the following: no ROSC leading to death, accidental displacement of the endotracheal tube or extubation, accidental displacement of venous or arterial catheters, use of nitric oxide for pulmonary hypertension, sepsis, pneumothorax, and intraventricular hemorrhage (grades 1–4) [38].

##### Data and Safety Monitoring Board

A DSMB will monitor the study to (1) protect all study patients, (2) safeguard the interests of all study patients, (3) monitor the overall conduct of the trial, (4) advise the investigators in order to protect the integrity of the trial, and (5) supervise the conduct and analysis of the interim analyses. The DSMB will receive regular reports from the trial on any injuries or adverse events, any developments that jeopardize the continued success of the trial, and data by which to accomplish the evaluation of predetermined early stopping rules. SAEs to be reported (mortality) will be sent within 72 h to the DSMB; reports of other/less serious adverse events and recruitment will be sent monthly; demographics and adverse events (including pneumothorax and intraventricular hemorrhage grade 3 or higher according to Papile [38]) will be included with the interim and final safety and efficacy analyses. The DSMB will perform interim safety analyses at 10%, 25%, and 50% of enrollment to review the primary outcome of ROSC, and SAEs. At the discretion of the DSMB further interim analyses can be requested.

Members of the DSMB are Myra Wyckoff (current Chair of the International Liaison Committee on Resuscitation, Neil Finer (a leading clinician-scientist in Neonatology), and Maryna Yaskina (Statistician, Biostatistics Unit, Women and Children’s Health Research Institute, University of Alberta). The members of the DSMB have no conflicts of interest and do not affiliate with the study sponsors.

##### Suspension or premature termination of the clinical investigation

The sponsor/principal investigator and the ethics committees can make decisions about trial discontinuation. If the trial is terminated or suspended, the parents of all trial participants will be informed, and appropriate follow-up will be ensured. If the sponsor/principal investigator terminates or suspends the trial, the relevant ethics committees will be provided with a detailed written explanation of the termination or suspension.

The sponsor/principal investigator can, upon completion of the analysis of the reason(s) for a suspension, decide to lift the suspension when the necessary corrective actions have been implemented. The investigators and ethics committees will be notified and provided with the relevant data supporting the decision.

##### Stopping rules

The DSMB will review the data at interim analyses at 10%, 25%, and 50% of enrollment. Predefined stopping rules will include:

1. An increased mortality in the CC + SI group by 25% compared to the 3:1 C:V group
2. Increase in rate of morbidities including pneumothorax, intraventricular hemorrhage, or a combination in the CC + SI group by 25% compared to the 3:1 C:V group
3. Bayesian posterior probability of CC + SI group being better than the control is < 0.5 or > 0.98.

##### Opt-out rule

In any cases where CPR is ongoing for 5 min, the clinical team must convert to the standard method of care using the 3:1 C:V ratio.

#### Data management

##### Data handling and archiving

Source data will be registered in the participant’s medical records/CRF and into the eCRF. A common web-based eCRF will be devised to enable a central database (Women and Children’s Health Research Institute, University of Alberta). Data entry into the central database and handling of medical records are the responsibility of the investigators. After the establishment of a “clean file,” the database will be locked. The data will be locked after completion of patient recruitment and data entry. After long-term follow-up data entry, this portion of the database will be locked. Data will be stored for statistical analysis at the Biostatistics Unit, Women and Children’s Health Research Institute, University of Alberta. The trial database will hereafter be kept according to the respective national laws. After the trial is finished, the data will be archived for 5 years according to Good Clinical Practice (GCP) guidelines. At each trial site the data flow will be monitored according to the GCP principles by a locally appointed external monitoring committee [39]. After completion of statistical data analysis, data will be pseudo-anonymized and stored at the University of Alberta.

##### Data protection

The investigator(s) will permit trial-related monitoring, audits, and regulatory inspection(s) by providing direct access to the source data and other relevant documents. Trial data will be handled according to regulations of the data protection agency in the respective countries.

#### Quality assurance

The trial will be carried out in accordance with the Declaration of Helsinki in its latest form and the International Conference on Harmonization Good Clinical Practice (ICH-GCP) guidelines [39].

#### Monitoring

The chief investigator consents to data evaluation being performed by the person in charge of monitoring in accordance with the monitoring plan, to ensure satisfactory data collection and adherence to the study protocol. Furthermore, the chief investigator states that he/she is willing to cooperate with this person and shall provide this person with all required information whenever necessary. This includes access to all documents related to the trial, including study-relevant medical files of patients in original form. The tasks of the investigator include maintenance of these patients’ medical files as comprehensively as possible; this includes information concerning medical history, accompanying diseases, inclusion in the trial, data about visits, results of investigations, dispensing of medication, and adverse events. The monitor will also be permitted to perform data evaluation and draw comparisons with the relevant medical files in accordance with the standard operating procedures and ICH-GCP guidelines at predetermined intervals [39] in order to ensure adherence to the study protocol and continuous registration of data. All original medical reports required as sources for the information given in the CRF or the database shall be inspected. The study participants will have given their consent to such inspection by signing the consent form. The person in charge of monitoring is obliged to treat all information as confidential and to preserve the basic claims of the study participants with respect to integrity and protection of their privacy.

## Potential significance

Morbidity and mortality rates are extremely high for newborns requiring CC. We believe the combination of simultaneous CC and SI during CPR has the potential to significantly improve ROSC and survival. In addition, we believe that CC + SI might improve respiratory and hemodynamic parameters and potentially minimize morbidity and mortality in newborn infants. In addition, this will be the first randomized controlled trial to examine CC in the newborn period.

## Trial status

At the time of this submission, this trial has been approved by the Human Research Ethics Board, University of Alberta, Edmonton, Canada and has recruited seven subjects at selected study sites so far.

## Additional file

Additional file 1: SPIRIT 2013 checklist: recommended items to address in a clinical trial protocol and related documents. (DOC 122 kb)

## Abbreviations

aEEG: Amplitude integrated electroencephalography
C:V ratio: Compression-to-ventilation ratio
CC + SI: Chest compression during sustained inflation
CC: Chest compression
CCaV: Continuous chest compression with asynchronous ventilations
CPR: Cardiopulmonary resuscitation
DBP: Diastolic blood pressure
DR: Delivery room
DSMB: Data and Safety Monitoring Board
ECO_2_: Exhaled carbon dioxide
eCRF: Electronic Case Report Form
GCP: Good Clinical Practice
IQR: Interquartile range
NIRS: Near-infrared spectroscopy
NNP: Neonatal Nurse Practitioner
PEEP: Positive end-expiratory pressure
PIP: Peak inflation pressure
RN: Registered Neonatal Nurse
ROSC: Return of spontaneous circulation
RRT: Registered Respiratory Therapist
SAE: Serious adverse event
SD: Standard deviation
SI: Sustained inflation
V_T_: Tidal volume
